# Extract of *Polygala tenuifolia* Alleviates Stress-Exacerbated Atopy-Like Skin Dermatitis through the Modulation of Protein Kinase A and p38 Mitogen-Activated Protein Kinase Signaling Pathway

**DOI:** 10.3390/ijms18010190

**Published:** 2017-01-18

**Authors:** Bongjun Sur, Bombi Lee, Ye Seul Yoon, Pooreum Lim, Riwon Hong, Mijung Yeom, Hyang Sook Lee, Hijoon Park, Insop Shim, Hyejung Lee, Young Pyo Jang, Dae-Hyun Hahm

**Affiliations:** 1 Acupuncture and Meridian Science Research Center, College of Korean Medicine, Kyung Hee University, Seoul 02447, Korea; surzeus@naver.com (B.S.); bombi@khu.ac.kr (B.L.); lpr911001@naver.com (P.L.); smsmjhjh@naver.com (R.H.); myeom@khu.ac.kr (M.Y.); erc633@khu.ac.kr (H.S.L.); acufind@khu.ac.kr (H.P.); ishim@khu.ac.kr (I.S.); hjlee@khu.ac.kr (H.L.); 2 Department of Life and Nanopharmaceutical Sciences, College of Pharmacy, Kyung Hee University, Seoul 02447, Korea; psycheb@naver.com (Y.S.Y.); ypjang@khu.ac.kr (Y.P.J.); 3 Department of Science in Korean Medicine, Graduate School, Kyung Hee University, Seoul 02447, Korea

**Keywords:** atopic dermatitis, immobilization stress, corticotrophin-releasing factor, *Polygala tenuifolia* Willd., trimellitic anhydride

## Abstract

Atopic dermatitis (AD) and stress create a vicious cycle: stress exacerbates atopic symptoms, and atopic disease elicits stress and anxiety. Targeting multiple pathways including stress and allergic inflammation is, therefore, important for treating AD. In this study, we investigated the remedial value of *Polygala tenuifolia* Willd. (PTW) for treating immobilization (IMO) stress-exacerbated atopy-like skin dermatitis and its underlying mechanism. Trimellitic anhydride (TMA) was applied to dorsal skin for sensitization and subsequently both ears for eliciting T-cell-dependent contact hypersensitivity in mice, which underwent 2 h-IMO stress and PTW administration for the latter 6 and 9 days in the ear exposure period of TMA, respectively. To elicit in vitro degranulation of human mast cell line-1 (HMC-1), 10 µM substance P (SP) and 200 nM corticotrophin-releasing factor (CRF) were sequentially added with 48 h-interval. PTW extract (500 µg/mL) was added 30 min before CRF treatment. IMO stress exacerbated TMA-induced scratching behavior by 252%, and increased their blood corticosterone levels by two-fold. Treatment with 250 mg/kg PTW significantly restored IMO stress-exacerbated scratching behavior and other indicators such as skin inflammation and water content, lymph node weights, and serum histamine and immunoglobulin E (lgE) levels. Furthermore, it also reversed TMA-stimulated expression of tumor necrosis factor (TNF)-α and interleukin (IL)-4 mRNAs in ear tissues. PTW significantly inhibited SP/CRF-stimulated degranulation of HMC-1 cells, subsequent tryptase secretion, and protein kinase A (PKA) activity. PTW also selectively inhibited p38 mitogen-activated protein kinase (MAPK) phosphorylation in SP/CRF-treated HMC-1 cells. PTW significantly inhibited HMC-1 cell degranulation and alleviated IMO stress-exacerbated atopic dermatitis symptoms by modulating the PKA/p38 MAPK signaling pathway.

## 1. Introduction

The incidence of atopic dermatitis (AD) is increasing worldwide, with a current prevalence rate of 20%–30% [1]. AD is most common in infants and children; however, the condition persists into adulthood in a minority of cases, affecting approximately 10% of the adult population, and its prevalence has increased in urbanized societies over recent decades [2]. In 95% of pediatric cases, AD symptoms occur before five years of age. However, adult onset of AD symptoms occurs in 15% of adult cases [3]. Adult AD generally has a more complex pathogenesis than does pediatric AD [4], and its causes include work-related stress, industrialization, urbanization, and pollution. Stress is a well-established trigger and aggravator of adult AD. Adult AD symptoms are exacerbated by a vicious cycle involving scratching, inflammation and stress.

Several studies suggest that stress triggers the release of corticotrophin-releasing factor (CRF) and substance P (SP) in the central and peripheral nervous systems. CRF and SP act on CRF receptors in the skin, causing the release of histamine and pro-inflammatory cytokines, such as tumor necrosis factor (TNF)-α and interleukin (IL)-4, and IL-6 [5]. Especially, a dysregulated type 2 T-helper (Th2) response is thought to be critical to the pathology of diseases including AD, which are characterized by Th2-dominated allergic inflammation. Th2-like immune responses mediated by IL-4 are important for the pathogenesis of atopic disorders because up-regulation of immunoglobulin E (IgE), one of the major causes of atopic inflammation, is regulated by IL-4, a representative Th2 cytokine. CRF may trigger mast cell (MC) activation directly or may act in synergy with SP to induce allergic skin inflammation, which aggravates AD [6]. MC-derived pro-inflammatory factors contribute to the pathogenesis of allergic or inflammatory skin diseases such as adult AD [7] Acute or chronic stress-induced CRF and SP release may trigger degranulation of MCs in mice [8]. CRF is involved in a number of intracellular signaling pathways [9]. In most cells, binding of CRF to the CRF1 receptor increases the activity of protein kinase A (PKA), which in turn phosphorylates and activates its downstream targets [10]. Furthermore, CRF receptor-mediated activation of mitogen-activated protein kinase (MAPK) signal transduction pathways has been reported [11], and the release of inflammatory mediators is considered to be mediated via intracellular signaling pathways including MAPKs [11]. Stimulation of the PKA/p38 MAPK pathway is crucial for CRF-mediated degranulation in human mast cell line-1 (HMC-1).

The roots of *Polygala tenuifolia*, a traditional Oriental medicine, have been widely reported to have multiple physiological roles and to produce a variety of biological effects, such as antioxidant, anticoagulant, antitumor, antiviral, analgesic, and anti-inflammatory activities, in the peripheral and central nervous systems [12,13,14]. At present, it is not known whether *Polygala tenuifolia* Willd. (PTW) can influence adult type-AD symptoms aggravated by stress in mice and if it does, whether the degranulation of MCs in skin tissues via stress-induced release of SP and CRF is involved. The present study thus aimed to evaluate the efficacy of PTW in reducing the stress-related exacerbation of AD symptoms using an in vitro MC degranulation assay and an in vivo trimellitic anhydride (TMA)-induced AD mouse model with immobilization (IMO)-aggravated stress to clarify the mechanism of action of PTW.

## 2. Results

### 2.1. Identification of Phytochemicals by Ultra Performance Liquid Chromatography (UPLC)-Electrospray Ionization (ESI)-Mass Spectrometry (MS)

**Figure 1:**
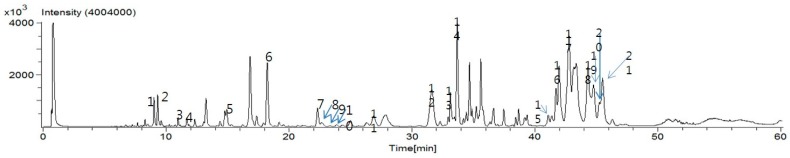
Ultra performance liquid chromatography (UPLC)–electrospray ionization (ESI)–mass spectrometry (MS) base peak chromatograms for compound profiles of *Polygala tenuifolia* Willd.

To characterize the phytochemicals in PTW, an ultra performance liquid chromatography (UPLC)–electrospray ionization (ESI)–mass spectrometry (MS) was performed. By virtue of the high resolution and high speed of UPLC, and accurate mass measurement by time-of-flight (TOF)-MS, a total of 21 compounds were identified from the PTW extract [15]. The liquid chromatography (LC)-MS total ion chromatogram of the PTW extract is shown in Figure 1. The retention time, observed mass, mass difference, and proposed compounds of 21 peaks are listed in Table 1.

### 2.2. Ear Manifestations and Hematoxylin-Eosin Histochemistry in Immobilization (IMO) Stress-Exacerbated Atopic Dermatitis (AD) Mice

**Figure 2:**
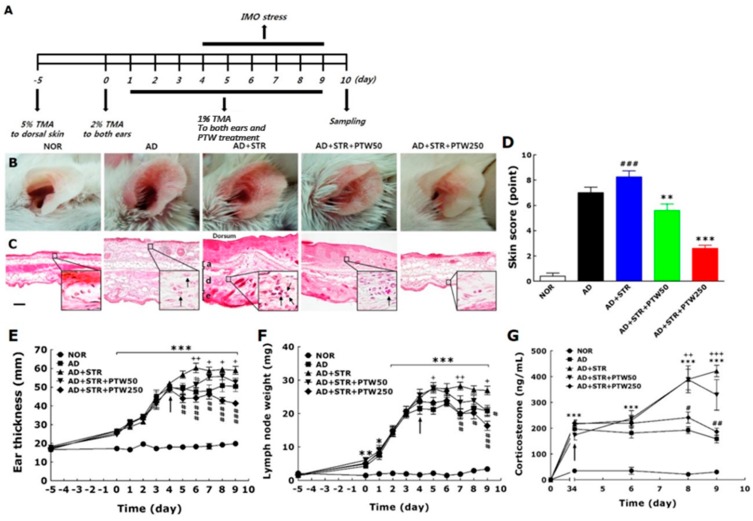

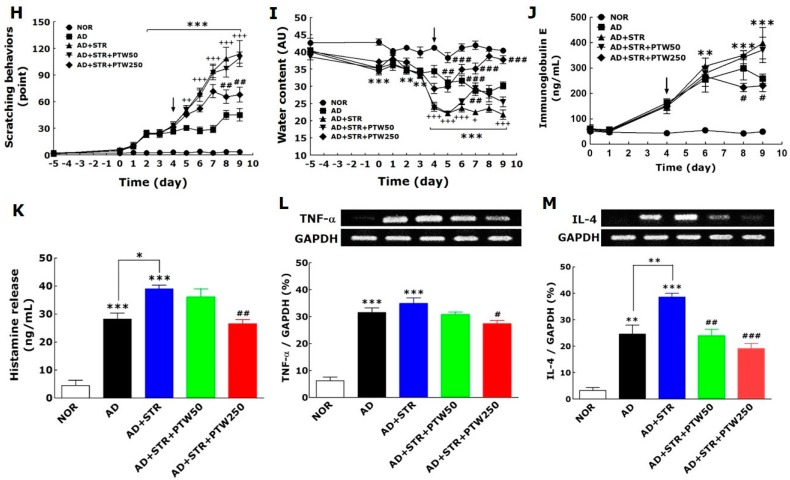
Inhibitory effect of *Polygala tenuifolia* Willd. (PTW) on immobilization (IMO) stress-exacerbated atopy-like skin symptoms in the mouse model of trimellitic anhydride (TMA)-induced contact hypersensitivity. Schematic diagram of the in vivo experimental schedule (**A**); and representative images of mouse ears (**B**) and their histological sections (**C**) are presented: a: auricular cartilage; d: dermis; e: epidermis in the vehicle-treated and IMO stress-exacerbated atopic group (AD + STR). Enlarged histological figures in black squares in the lower right corners indicate the infiltration of inflammatory cells in dermis layers. Arrows in the squares indicate immune cells such as neutrophil or eosinophil. Scale bar in non-treated normal group (NOR) indicates 100 µm. Skin score (*n* = 10) (**D**) indicating atopic dermatitis severity based on the mouse ear images, ear thickness (*n* = 10) (**E**); lymph node weight (*n* = 10) (**F**); blood corticosterone levels (*n* = 5) (**G**); scratching behavior (*n* = 10) (**H**); skin water content (*n* = 10) (**I**); and serum levels of immunoglobulin E (IgE) (*n* = 5) (**J**); and histamine (*n* = 5) (**K**) in each group are also presented in the graphs. Arrows in E, F, G, H, I and J indicate the initiation of IMO stress on day four. The mRNA expression levels of tumor necrosis factor (TNF)-α (*n* = 5) (**L**) and interleukin (IL)-4 (*n* = 5) (**M**) in ear skin tissues are also presented in polymerase chain reaction (PCR) band images and bar graph. Ear tissues were collected from five mice randomly selected in each group. AU, arbitrary unit; PTW, *Polygala tenuifolia* Willd.; TMA, trimellitic anhydride; IMO, immobilization; GAPDH, glyceraldehyde 3-phosphate dehydrogenase. * *p* < 0.05, ** *p* < 0.01, and *** *p* < 0.001 vs. the NOR group; ^#^ *p* < 0.05, ^##^ *p* < 0.01, and ^###^ *p* < 0.001 vs. the AD + STR group; ^+^ *p* < 0.05, ^++^ *p* < 0.01, and ^+++^ *p* < 0.01 vs. the vehicle-treated and TMA-induced atopic group (AD).

In order to verify the effect of PTW on stress-aggravated atopic symptoms, macroscopic clinical signs, morphological changes, ear thickness, and lymph node weights were measured in mice with or without PTW administration. Apparent skin symptoms, such as edema, crusting, and excoriation, were exacerbated by immobilization (IMO) stress, as indicated in the vehicle-treated and IMO stress-exacerbated atopic (AD + STR) group being compared with the vehicle-treated and TMA-induced atopic (AD) and non-treated normal (NOR) groups (Figure 2B). Morphological changes in inflamed skin and cutaneous cell infiltration were also characterized by histological observation (Figure 2C). Noticeable swelling and inflammation, indicated by increases in the thicknesses of the epidermal and dermal layers compared with the AD group, were observed in the AD + STR group. However, in the AD + STR + PTW250 group, inflammation of the epidermis and dermis were significantly reduced compared with the AD + STR group. Their histological shapes were similar to those in the NOR group. Skin score quantifying the severity of atopic dermatitis symptoms in the AD + STR group was also increased by 15.2% compared with the AD group and 94.8% compared with the NOR group (Figure 2D). This IMO stress-induced exacerbation of atopic symptoms was dose-dependently suppressed by PTW administration, as observed in the AD + STR + PTW50 and AD + STR + PTW250 groups. In the present study, ear skin thickness was used as an indicator of cutaneous inflammation induced by TMA + IMO treatment. In the AD + STR + PTW250 group, ear thickness was reduced by 20% on day 9 (*p* < 0.001) compared with the AD + STR group (*p* < 0.01 on days five and seven; *p* < 0.001 on days six, eight, and nine) (Figure 2E). In mice with IMO stress-exacerbated AD, proliferation of lymph node cells and infiltration of various immune cells into the lymph nodes caused a significant increase in lymph node weight (Figure 2F). PTW administration also inhibited the increase in lymph node weight in a dose-dependent manner, showing a maximum inhibition of 63.4% compared with the AD + STR group at a dose of 250 mg/kg PTW (*p* < 0.05 on day eight; *p* < 0.01 on day seven; *p* < 0.001 on day nine). In order to analyze the neuroendocrine response to IMO stress applied to the mice underwent TMA exposure, blood corticosterone levels were compared between groups during repeated exposure to IMO stress from day four to nine. Blood corticosterone levels in the AD group rapidly increased to a concentration of ~200 ng/mL and were further increased by IMO stress, reaching over 400 ng/mL by day nine (Figure 2G). Compared with mice in the AD group, the corticosterone level in mice in the AD + STR group increased significantly to 622.19%, (*p* < 0.001 on days four, six, eight, and nind). This indicated that repeated IMO stress from days 4–9 was sufficiently stressful to aggravate TMA-induced atopic symptoms of AD mice. Daily administration of 250 mg/kg PTW to the AD + STR + PTW250 group significantly inhibited the IMO stress-induced increase in the corticosterone level in the blood, restoring it to almost the same level as in atopic mouse without stress in the AD + STR group (*p* < 0.05 on day eight and *p* < 0.01 on day nine). The AD + STR + PTW50 group treated with 50 mg/kg PTW showed little difference, as compared with the AD + STR group.

### 2.3. Scratching Behavior, Skin Water Content, and Serum Immunoglobulin E (IgE) and Histamine Levels in IMO Stress-Exacerbated AD Mice

When TMA only or TMA + IMO stress were applied sequentially to the mice, they vigorously scratched their lesional skins with their hind paws. Scratching behavior in the AD + STR group was markedly exacerbated starting from day two, and on day nine it became aggravated by 252% compared with that in the AD group (Figure 2H). Scratching behavior was reduced significantly in the AD + STR + PTW250 group compared with the AD + STR group (*p* < 0.01). The inhibition of scratching behavior was stronger in the AD + STR + PTW250 group than in the AD + STR + PTW50 group. Epidermal dysfunction, indicated by reduced water content of skin, was associated closely with scratching behavior. As shown in Figure 2I, skin water content declined significantly in the AD + STR group in a time-dependent manner. After the experiment, the water content was 21.8 ± 1.4 in the AD + STR group, compared with 40.4 ± 0.4 in the NOR group (*p* < 0.01 on day five; *p* < 0.001 on days 6–9).

An increase in serum immunoglobulin E (IgE) levels is an important measure of AD symptomatology. A noticeable increase in the serum IgE level was observed in the AD + STR group, as compared with the NOR group, and even with the AD group in spite of statistical insignificancy (Figure 2J). As expected, PTW administration reduced serum IgE levels. In the AD + STR + PTW250 group, the IgE level was reduced significantly on days eight and nine (*p* < 0.05), while there was no significant decrease of the IgE level in the AD + STR + PTW50 group. Histamine, the principal component of mast cell granules, plays a key role in exerting many effects related to the immediate phase of allergic inflammation, including vasodilation in AD-like diseases. The histamine levels increased significantly in the AD + STR group compared with those in the AD group on day nine (Figure 2K). After the experiment, the histamine level was 38.9 ± 1.3 in AD + STR group, compared with 28.1 ± 1.9 in SD group. The histamine level was significantly reduced in the AD + STR + PTW250 group compared with the AD + STR group (*p* < 0.05).

### 2.4. Tumor Necrosis Factor (TNF)-α and Interleukin (IL)-4 mRNAs in IMO Stress-Exacerbated AD Mice

We investigated the effects of PTW on the mRNA expression levels of TNF-α and IL-4, important cytokines in the pathogenesis of AD, in mice with IMO stress-exacerbated AD. The expression level of IL-4 mRNA increased significantly in the AD+STR group (57%, *p* < 0.05) compared with SD group despite little difference in case of TNF-α (24.3%) (Figure 2L,M). The expression levels of both cytokines were reduced after PTW administration in a dose-dependent manner. The AD + STR + PTW250 group showed 27.4 ± 1.2 (7.5%) and 19.1 ± 1.9 (19.5%) inhibition of TNF-α and IL-4 mRNA expression levels, respectively (*p* < 0.05 and *p* < 0.001). Statistical differences between groups were identified using *t*-test, one-way ANOVA, and Tukey’s post hoc test.

### 2.5. Toluidine Blue Staining and Tryptase Activity in Human Mast Cell Line-1 (HMC-1)

**Figure 3:**
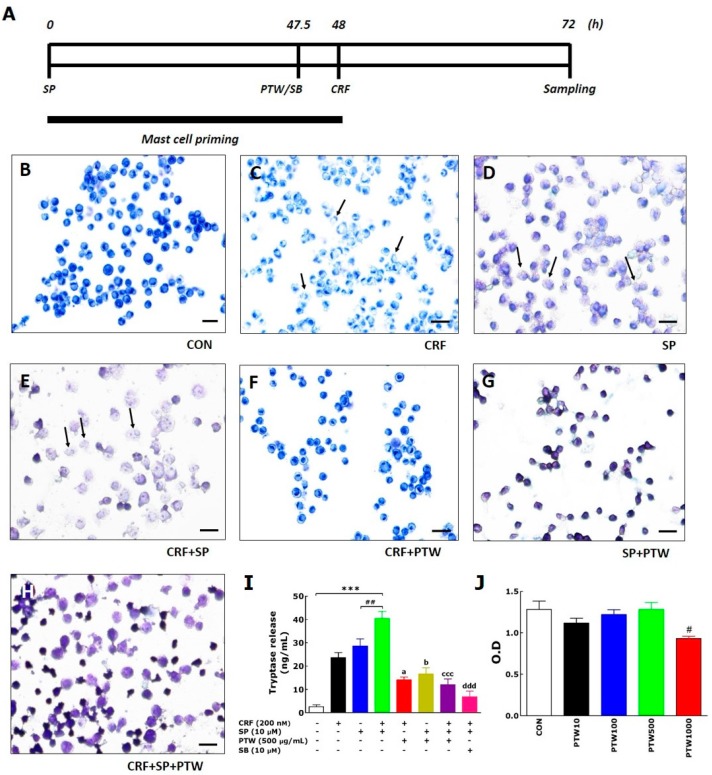
Schematic diagram showing *in vitro* experimental schedule (**A**) and representative images of toluidine blue-stained HMC-1 cells treated with vehicle as a control (CON, (**B**)); corticotrophin-releasing factor (CRF, (**C**)), substance P (SP, (**D**)); CRF + SP (**E**); CRF + *Polygala tenuifolia* Willd. (PTW, (**F**)); SP + PTW (**G**) and CRF + SP + PTW (**H**); their tryptase levels (**I**) and cell viability (**J**) measured in the culture medium during degranulation. In the experiments of (**B**–**D**), cells were harvested for assay 24 h after vehicle (medium, CON), CRF and SP treatments, respectively. In (**F**–**H**), PTW was added 30 min before SP and/or CRF treatment, and the cells were harvested 24 h after SP or CRF treatment. The cells in H were harvested according to the in vitro experimental schedule in A with PTW treatment. Arrows in (**C**–**E**) indicated the degranulated cells of HMC-1. CRF: corticotropin-releasing factor; SP: substance P; SB: SB203580; PTW: *Polygala tenuifolia* Willd.; O.D: optical density. SB20358 (10 µM), a p38 mitogen-activated protein kinase (MAPK) inhibitor, was used as a positive control of inhibiting HMC-1 degranulation. Plus (+) and minus (−) in the X-axis description of I indicate ‘treated’ and ‘non-treated’, respectively. Scale bar = 200 μm. *** *p* < 0.001 vs. non-treated HMC-1 cells (CON); ^##^ *p* < 0.01 vs. SP-treated HMC-1 cells; ^a^ *p* < 0.05, ^b^ *p* < 0.05, ^ccc^ *p* < 0.001, and ^ddd^ *p* < 0.001 vs. CRF + SP-treated HMC-1 cells; ^#^ *p* < 0.05 vs. vehicle-treated naïve HMC-1 cells (CON).

To examine the effect of PTW on intracellular signaling related to mast cell degranulation, CRF (200 nM) was added to HMC-1 cells after pre-treatment with SP (10 µM) for 48 h. PTW extract (500 µg/mL) was applied 30 min before HMC-1 cells were treated with CRF (Figure 3A). Sequential application of SP and CRF induced degranulation of HMC-1 cells causing the production and release of histamine. Whereas no morphological change was observed in non-treated HMC-1 cells, noticeable changes were observed in HMC-1 cells stimulated with CRF and SP, individually or together with 48 h-interval (Figure 3B–E). Notably, combined CRF + SP treatment caused maximum degranulation in HMC-1 cells. Treatment with PTW reduced degranulation in HMC-1 cells treated with SP and/or CRF, similar to that in non-treated HMC-1 cells (Figure 3F–H). Tryptase were also released in to the media by the treatment with CRF (23.6 ± 2.2), SP (28.6 ± 3.1) or CRF + SP (40.4 ± 3.0), whereas tryptase levels remained low in non-treated HMC–1 cells (CON, 2.6 ± 0.8) (Figure 3I). Moreover, PTW extract significantly suppressed tryptase release in HMC-1 cells (CRF + PTW, 14.0 ± 1.3; SP + PTW, 16.6 ± 2.6; CRF + SP + PTW, 12.0 ± 2.5). The PTW-induced suppression of tryptase release was most remarkable in CRF + SP + PTW-treated HMC-1 cells than in other conditions (*p* < 0.001). Interestingly, the final levels of tryptase activities in the CRF + PTW, SP + PTW and CRF + SP + PTW groups were all similar. SB203580 (SB, 10 µM) was used as a positive control in the current study. In order to determine the appropriate concentration of PTW at which cell growth of HMC-1 is not affected, cell viability was assessed using the MTT assay 24 h after PTW treatment. As shown in Figure 3J, PTW extract up to 500 µg/mL did not exhibit any significant cytotoxicity; however, the treatment with greater than 1000 µg/mL resulted in the significant cytotoxicity.

### 2.6. Activities of Protein Kinase A (PKA) and Protein Kinase C (PKC) in HMC-1 Cells

**Figure 4:**
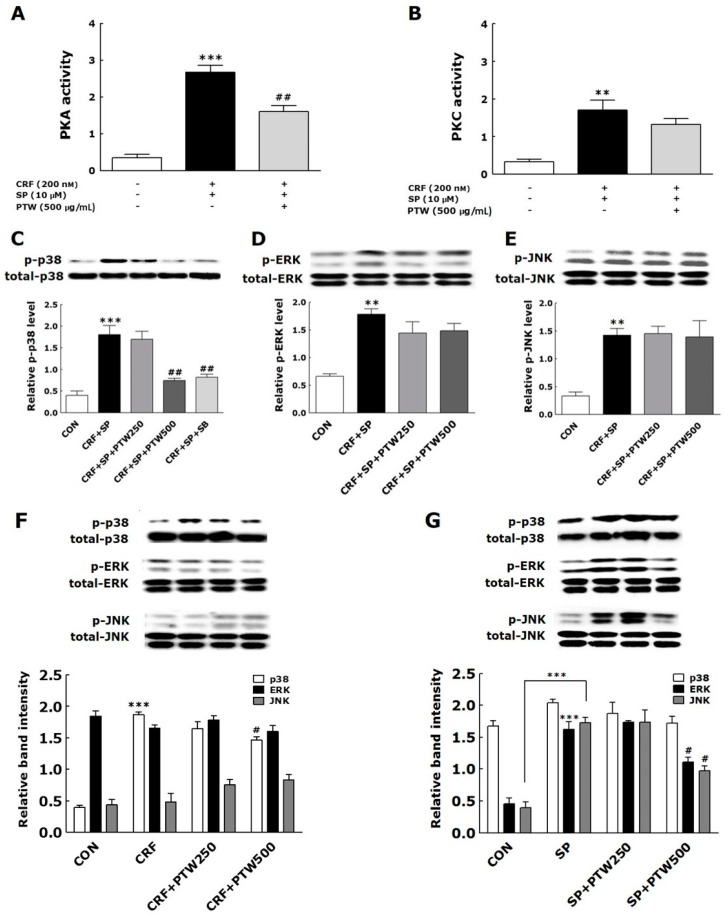
Effect of PTW extract (250 or 500 µg/mL) on PKA (**A**) and PKC (**B**) activities, phosphorylation of p38 (**C**), ERK (**D**) and JNK (**E**) MAPKs in HMC-1 cells previously stimulated by sequential treatments with SP and CRF. PTW efficacy on phosphorylation of MAPKs was also examined in HMC-1 cells stimulated by CRF (**F**) only or SP (**G**) only. CRF: corticotropin-releasing factor; SP: substance P; SB: SB203580; HMC: human mast cell; PTW: *Polygala tenuifolia* Willd.; PKC: protein kinase C; PKA: protein kinase A; ERK: extracellular signal-regulated kinase; JNK: c-Jun N-terminal kinase; MAPK: mitogen-activated protein kinase. SB20358 (10 µM), a p38 mitogen-activated protein kinase (MAPK) inhibitor, was used as a positive control of inhibiting p38 phosphorylation in HMC-1 cells. Plus (+) and minus (−) in the X-axis description of A and B indicate “treated” and “non-treated”, respectively. ** *p* < 0.01 and *** *p* < 0.001 vs. non-treated HMC-1 cells (CON); ^#^ *p* < 0.05 and ^##^ *p* < 0.01 vs. CRF + SP-treated HMC-1 cells.

In mast cells, sequential treatment with SP and CRF increase the activities of PKA and PKC, which phosphorylate and activate downstream targets [16,17]. In the present study, application of SP and CRF to HMC-1 cells also resulted in increased PKA and PKC activities compared with those in non-treated HMC–1 cells in which PKA and PKC activities were not observed (Figure 4A,B). The activities of PKA and PKC in CRF + SP-treated HMC–1 cells were 2.7 ± 0.2 and 1.7 ± 0.3, respectively. PTW significantly decreased PKA activity by 1.6 ± 0.2 (*p* < 0.01) despite an insignificant decrease of PKC activity by PTW.

### 2.7. MAPK Signaling Pathways in HMC-1 Cells

MAPK pathways have important roles as signaling mediators of cellular responses to extracellular signals associated with mast cell degranulation in AD [16]. We analyzed the phosphorylation of p38, extracellular signal-regulated kinase (ERK), c-Jun N-terminal kinase (JNK) after the sequential treatments of HMC-1 cells with SP and CRF to induce degranulation, and then the following treatment with PTW extract. Sequential treatments of HMC-1 cells with SP and CRF markedly increased the phosphorylation levels of p38. ERK and JNK. In non-treated HMC–1 cells (CON), phosphorylation of any MAPKs was not observed. Treatment with 500 µg/mL PTW significantly inhibited SP + CRF-induced phosphorylation of p38 (Figure 4C). However, no significant inhibition was observed under the treatment of 250 µg/mL PTW. The treatments of PTW extracts did not influence the phosphorylation levels of ERK and JNK, previously activated with SP and CRF, irrespective of treatment concentration (Figure 4D,E). We also tested kinase phosphorylation using SB, a specific p38 MAPK inhibitor as a control. Phosphorylation of p38 was completely blocked by SB (10 µM) (Figure 4C). Taken together, these results indicate that ERK and JNK are not involved in CRF + SP-induced degranulation of mast cells, whereas p38 MAPK is critical to this process.

Similarly, only p38 was phosphorylated in CRF-stimulated HMC-1 cells (Figure 4F). In the HMC-1 cells harboring CRF-induced phosphorylation of p38 MAPK, PTW extract slightly inhibited its phosphorylation level at the concentration of 500 µg/mL. Conversely, in the case of pre-treatment of SP, ERK and JNK were markedly phosphorylated (Figure 4G), while p38 was not. PTW also inhibited SP-induced phosphorylation of ERK and JNK only at 500 µg/mL.

## 3. Discussion

PTW has long been recognized as an invaluable medicinal source for treating and preventing various human diseases including inflammatory, allergic and mental diseases. Due to its property of multiple targeting and improving various mental dysfunction based on traditional medicine theory, it is possible that PTW has medicinal properties, which may lead to the development of new medicines for treating stress-sensitive allergic skin diseases including AD in humans. We previously reported an anxiolytic activity of PTW extract through analyzing stress–induced anxiety–like behavior in the elevated plus maze following repeated IMO stress in mice [13]. Connected to this result, current study investigated the efficacy and mechanisms underlying the anti-stress and anti-AD effects of PTW using the IMO stress-exacerbated AD mouse model.

As an in vitro model, we exploited SP/CRF-double treated HMC-1 cells to mimic stress-associated AD condition. In the MCs, only the individual application of either CRF or SP can induce degranulation, but this phenomenon is a simple allergic response in the AD mouse. However, application of CRF to HMC-1 cells after SP pretreatment can have a larger influence on allergic disease, particularly via hypersensitive allergic and inflammatory responses in the MCs [16,17]. In terms of clinical study, the application of SP and CRF to HMC-1 is certainly a more viable approach for patients with stress-associated AD. Moreover, MC activation can induce the release of histamine and pro-inflammatory cytokines as well as scratching behavior in mice [18]. Using SP/CRF-double treated HMC-1 cells, we tested anti-MC degranulation and anti-inflammation activities of several extracts of traditional medicine which has been known to be effective to treat various skin dysfunction and eventually isolated PTW among them (data not shown).

The onset of adult AD is associated with many factors such as psychological stress, industrialization, urbanization, and pollution, all of which steadily exacerbate the condition. Of these factors, stress is the single most important aggravator of atopic symptoms [19]. Atopic dermatitis (AD) and stress, thus, can create a vicious cycle: stress exacerbates atopic symptoms and subsequently atopic symptoms elicit stress and anxiety. To establish the in vivo animal model in the current study, mice with TMA-induced AD were exposed to IMO stress repeatedly to worsen the atopic skin conditions. In a preliminary study, mice subjected to chronic foot shock stress exhibited similar levels of scratching behavior compared with mice with TMA-induced AD (data not shown). However, in the present study, AD mice exposed to repeated IMO stress showed a remarkable increase in scratching behavior compared AD mice without stress. Repeated IMO stress that worsened AD symptoms also significantly increased the releases of corticosterone, histamine, and IgE, suggesting that these factors may be of use when diagnosing and treating stress-associated AD. Although this model, as described above, is limited due to its lack of de novo induction of all AD-like skin lesions, it focuses on the IMO stress-induced exacerbation of AD. Over the past several decades, there has been a concerted effort to create animal models of AD.

Itching is one of the most relevant symptoms associated with AD-like skin diseases and can be exacerbated steadily by inflammation, cancer, metabolic diseases, infection, psychiatric diseases, drug abuse, stress, and other factors [20]. An inflammatory skin environment lowers the threshold for itch stimuli and causes sensitization to itching. Itching is the chief concern in an AD animal, because scratching behavior exacerbates skin symptoms of AD such as thickened, cracked, dry, scaly skin, and inflammation. In the present study, the administration of PTW dramatically alleviated scratching behavior. However, interestingly, it did not take the scratching behavior down the level of TMA-induced AD, which means that PTW only influenced the itching symptoms aggravated by IMO stress, although it has been reported that PTW and some of its components have anti-inflammatory activity through nuclear factor (NF)-κB pathway [21].

Over the past several decades, inflammation and the immune response have been implicated as key players in the pathogenesis of AD. In fact, the cytokine production in response to AD is widely recognized as a central mediator of cutaneous AD lesions [22]. TNF-α has a wide variety of biological effects in humans, while IL-4 can amplify IgE-induced signals in MCs via up-regulation of Fc ε receptor 1 (FcεR1) expression on the cell surface and provides a receptive environment for eosinophil recruitment due to its presence in local tissues [23]. In the present study, it was observed that IL-4 is a more damaging and important factor than TNF-α in stress-associated AD, and a greater decrease in IL-4 mRNA expression relative to TNF-α by PTW treatment. Based on these results, it can be suggested that PTW can more efficiently alleviate stress-exacerbated allergic conditions than the simple inflammatory condition and, thus, may have a more powerful therapeutic role in adult type-AD.

In a previous study, various pathophysiological processes such as MC degranulation, inflammation and proopiomelanocortin gene expression were initiated by activation of PKA signaling through CRF–receptor 1 on the skin cell surface [16,17,24,25]. It was found that the treatments of CRF and SP activated PKA and PKC via CRF receptor 1 (CRFR1) and neurokinin 1 receptor (NK1R), respectively, in HMC-1 cells [9,16]. In the present study, PTW also inhibited PKA activity but not significantly for PKC. Since PTW extract was added to HMC-1 cells between SP and CRF treatment, that is, 48 h after SP treatment and 30 min before CRF treatment, PKA may not be sufficiently activated unlike PKC at the moment of PTW treatment. If PTW can modulate PKC signaling mainly through the inhibition of phosphorylation level of PKC protein, it hardly affected PKC activity after SP treatment. It was recently reported that the development of pharmacological inhibitors targeting MAPKs may be an attractive strategy for the treatment of allergic diseases [26]. Azzolina et al. [27] reported that the induction of TNF-α expression and histamine exocytosis following the exposure of rat peritoneal mast cells to SP requires activation of the p38 and JNK MAPK pathways. CRF induces cell proliferation and TNF-α release in the in vitro rat microglia cells via the activation of ERK and p38 MAPK and down-regulates IL-18 expression in human HaCaT keratinocytes via activation of the p38 MAPK pathway [28]. In the present study, PTW prevented the phosphorylation of p38 MAPK but did not affect phosphorylation of JNK and ERK in HMC-1 cells stimulated with CRF only or both SP and CRF. These findings suggested that CRF rather than SP selectively activated p38 signaling among MAPKs, and PTW could inhibit such activation that leads to degranulation of HMC-1. Under the condition of pre-primed HMC-1 cells with SP, the CRF-induced activation of p38 MAPKs and its inhibition by PTW extract were more pronounced than under the condition of non-treatment of SP.

In vitro and in vivo experiments showed that the IMO stress-exacerbated AD mouse model is valid and that PTW inhibited mast cell degranulation in HMC-1 cells. Moreover, PTW significantly reduced stress-induced exacerbation of AD via modulation of p38 MAPK cell signaling rather than by affecting intrinsic signaling of atopic inflammation itself. Thus, PTW may be a useful treatment for stress-exacerbated AD. Moreover, our results provide a clue for explaining and possibly developing new therapeutic approaches for the treatment of inflammatory and allergic skin diseases, especially those affected by psychological stress.

## 4. Experimental Procedures

### 4.1. Animals and Cell Line

Twelve-week-old male Balb/c mice weighing 28–30 g were obtained from Samtaco Animal Co. (Osan, South Korea). The mice were housed in a limited access rodent facility with up to five mice per polycarbonate cage under the temperature at 22 ± 2 °C, the relative humidity at 55% ± 15% and artificial light for 12 h each day. The animal experiments were conducted in accordance with the National Institutes of Health *Guide for the Care and Use of Laboratory Animals* (NIH Publications No. 80-23), revised in 1996, and were approved by the Kyung Hee University Institutional Animal Care and Use Committee. HMC-1 cell line was obtained from the Korean Collection for Type Cultures (Daejon, South Korea). HMC-1 cells were grown in a 5% CO_2_/95% air humidified atmosphere at 37 °C in Iscove’s Modified Dulbecco’s Medium (Life Technologies, Carlsbad, CA, USA) supplemented with 10% (*v/v*) heat-inactivated fetal bovine serum (Welgene, Seoul, South Korea) and a 1% (*v/v*) mixture of penicillin and streptomycin (Sigma-Aldrich Chemical Co., St. Louis, MO, USA).

### 4.2. Reagents

CRF, SP, SB203580 (SB), TMA (98%) and isopropyl myristate (98%) were purchased from Sigma-Aldrich Chemical Co., and dissolved in deionized H_2_O or acetone (Merck, Darmstadt, Germany) immediately before use. Dried roots of PTW were obtained from an Oriental drug store (Dongwoodang Pharmacy Co., Ltd., Yeongcheon, Republic of Korea). The voucher specimen (No. D0801130PTW) was deposited at the herbarium located in the college of Korean Medicine, Kyung Hee University. To obtain a boiling water extract of PTW, 120 g PTW was immersed in distilled water, heated at 100 °C, concentrated using a rotary evaporator (Rotavapor R-124, Buchi, Flawil, Switzerland) and lyophilized using a freeze dryer (EYELA^®^; Tokyo Rikakikai Co., Ltd., Tokyo, Japan). The powder form of PTW extract (yield 14.16%) was used in every experiment after dilution in deionized H_2_O.

### 4.3. UPLC-ESI-MS

PTW granules (30 mg) were re-extracted in 1 mL methanol by sonication for 1 h at 50 °C. In order to remove the precipitate, the solution was centrifuged. After centrifugation, the supernatant was filtered through a 0.2 µm NORM–JECT syringe filter (Whatman International Ltd., Maidstone, Kent, UK) before injecting into an UPLC instrument. The UPLC instrument was an ACQUITY UPLC H-Class System operated using Empower 3 software (Waters, Milford, MA, USA). The PDA detector recorded data between 210 and 400 nm. The Brownlee SPP C18 column (3.0 × 100 mm, 2.7 µm) (PerkinElmer, Wellesley, MA, USA) was selected for the UPLC analysis. The monitoring wavelength of detector was set to 300 nm. The mobile phase was methanol acidified with formic acid (0.5%, solvent A), and water acidified with formic acid (0.5%, solvent B). The gradient program was 0–5 min, 2% solvent A; 5 min, 20% solvent A; 10 min, 30% solvent A; 18 min, 38% solvent A; 20 min, 40% solvent A; 30 min, 40% solvent A; 32 min, 50% solvent A; 38 min, 65% solvent A; 48 min, 70% solvent A; 50 min, 100% solvent A. The flow rate was maintained at 0.5 mL/min using a splitter. The injection volume was 2 µL. An AccuTOF^®^ single-reflectron-TOF-MS equipped with an electrospray ionization (ESI) source (JEOL USA, Inc., Peabody, MA, USA) was operated using MassCenter version 1.3.7b (JEOL USA, Inc., Peabody, MA, USA). In the negative ion mode, the atmospheric pressure interface potentials were typically set to the following values: orifice 1 = −90 V; ring lens and orifice 2 = −15 and −10 V, respectively. The ion guide potential and detector voltage were set to 2000 V and 2300 V, respectively. ESI parameters were set as follows: needle electrode = 2000 V; nebulizer and desolvating gas = nitrogen gas (flow rate 1 and 3 L/min, respectively); desolvating chamber temperature = 250 °C, orifice 1 temperature = 80 °C. Mass scale was calibrated using the YOKUDELNA calibration kit (JEOL Ltd., Tokyo, Japan) to achieve accurate mass measurements and calculations of elemental composition. MS acquisition was achieved using an *m/z* scan range of 100 to 2000.

### 4.4. Experimental AD Models and Drug Treatment

For the in vitro model of stress-associated AD, HMC-1 cells were washed with Dulbecco’s phosphate-buffered saline (DPBS; Gibco, Grand Island, NY, USA) and suspended in the culture medium. HMC-1 cells (2 × 10^5^ cells/200 µL/well) were plated in 96-well flat bottomed Falcon cell culture plates from Becton Dickinson (Franklin Lakes, NJ, USA) and subsequently incubated with 10 µM SP for 48 h at 37 °C in a 5% CO_2_ incubator. Next, 200 nM CRF was added, and then the cells were incubated for another 24 h. The plates were then centrifuged [16,17]. PTW (250 or 500 µg/mL) or SB (10 µM) was added 30 min before 200 nM CRF treatment. For in vivo experiments, AD-like skin lesions were generated by repeated application of TMA to both sides of both ears of Balb/c mouse [29]. Sensitization was induced by application of 5% TMA to dorsal skin, followed by application of 2% TMA to both ears on day 0. Then, 1% TMA was applied once a day from days one to nine. After each application of 1% TMA, the mice underwent 2 h of IMO stress from days four to nine. PTW (50 or 250 mg/kg) was administered orally 30 min before daily application of 1% TMA from days one to nine. IMO stress was achieved by placing the animals for 2 h in a transparent plastic 50 mL-Falcon tube (Greiner, Frickenhausen, Germany) with a diameter of 3 cm and a handmade ventilation hole for breathing.

### 4.5. Experimental Groups

The mice were randomly divided into five experimental groups of ten animals each as follows: non-treated normal group (NOR, *n* = 10); vehicle-treated and TMA-induced atopic group (AD, *n* = 10); vehicle-treated and IMO stress-exacerbated atopic group (AD + STR, *n* = 10); 50 mg/kg PTW-treated abnd IMO stress-exacerbated atopic group (AD + STR + PTW50, *n* = 10); 250 mg/kg PTW-treated and IMO stress-exacerbated atopic group (AD + STR + PTW250, *n* = 10).

### 4.6. Ear Skin Manifestation, Histochemistry, and Scratching Behavior

The mouse ear skins in each experimental group were photographed using a digital camera (Canon 20D; Canon Inc., Tokyo, Japan) to analyze clinical appearance. Atopic symptoms were evaluated at day nine by scoring scaling and dryness, hemorrhage and excoriation, and edema and redness, and by then calculating the sum of the individual symptom scores for both ears, graded as 0 (no symptoms), 1 (mild), 2 (moderate), or 3 (severe). The total score for each animal ranges from zero to nine (22). Ear thickness was measured once a day using a dial thickness gauge (Ozaki Seisakusho Co., Tokyo, Japan). The auricular lymph node weight was measured daily using a digital balance (Mettler Toledo Co., Greifensee, Switzerland). Water content of the stratum corneum in the skin epidermis was determined by measuring electrical capacitance of skin using a Corneometer^®^ CM825 (Courage and Khazaka, Cologne, Germany), with values indicated in arbitrary units (AU). The skin area with the highest coefficient of insulation was chosen for the measurement. The average of three measurements for each area was calculated. For hematoxylin–eosin histochemistry, five mice from each group were anesthetized with sodium pentobarbital (50 mg/kg, intraperitoneal), and their ear skin tissues were collected. The tissues were fixed in 10% paraformaldehyde overnight, dehydrated with 99% ethanol, embedded in paraffin, sectioned to a thickness of 6 µm using a microtome (Finesse 325; Thermo Scientific, Rockford, IL, USA), and mounted onto slides. Before staining, the tissue sections on the slides were deparaffinized. Subsequently, the tissues were stained with hematoxylin (Merck Co., Darmstadt, Germany) and 1% eosin (Sigma-Aldrich Chemical Co., St. Louis, MO, USA), air-dried, and cover-slipped for microscopic observation. All slides were photographed at 100× magnification using a microscope equipped with a camera (BX51; Olympus Ltd., Tokyo, Japan) and analyzed using DP2-BSW software (Olympus Ltd.) by an observer blinded to the experimental groups. All images were selected from at least three different ear skin tissue images per mouse. The behavior of all mice was recorded using a video camera for 10 min under strictly quiet conditions. During the video recording, the number of scratching episodes in 10 min was counted by investigators who were blinded to the group classification and drug treatment given. The mice scratched several times with their hind paws for 1 s in general, and these movements were counted as one episode of scratching. 

### 4.7. Cell Viability

Cell viability was determined by using an EZ-Cytox^®^ cell viability assay kit including water soluble tetrazolium salt (WST)-1 (DaeilLab Service Co., Seoul, South Korea). Briefly, HMC-1 cells were cultured overnight at a density of 2.5 × 10^5^ cells per well in 96-well plates with low serum (1% FBS), followed by treatment with various concentrations up to 1000 µg/mL of AC extract. After 24 h, 10 µL WST-1 reagent was added to each well. After 1 h-incubation at room temperature, the plates were read at 450 nm using a microplate reader (Molecular Devices Co., Sunnyvale, CA, USA).

### 4.8. Enzyme-Linked Immunosorbent Assay (ELISA)

For histamine analysis, blood samples were harvested from the retro-orbital plexus from mice under non-anesthetized conditions on the day of killing using a capillary. Serum was obtained by centrifugation at 6500 rpm for 20 min and stored at −70 °C until use. Histamine, IgE and corticosterone levels were measured using ELISA kits for histamine (Labor Diagnostika, Nord GmbH and Co., KG, Nordhorn, Germany), IgE (Bethyl Laboratories Inc., Montgomery, TX, USA) and corticosterone (ABcam, Cambridge, MA, USA), respectively, according to the manufacturers’ instructions. All reaction products were measured at 450 nm using an ELISA reader (MultiRead 400; Anthos Co., Vienna, Austria), and their amounts were calculated in ng/mL from standards.

### 4.9. Reverse Transcription Polymerase-Chain Reaction (RT-PCR)

Five mice from each group were anesthetized deeply with sodium pentobarbital (50 mg/kg, i.p.), and their ear tissues were collected. Total RNAs were isolated from the ear tissues (50−100 mg) or harvested HMC−1 cells using TRIzol^®^ reagent (Invitrogen Co., Carlsbad, CA, USA), according to the manufacturer’s instructions. Complementary DNA (cDNA) was synthesized from total RNA using PrimeScript^TM^ reverse transcriptase (Takara Co., Shiga, Japan). The expression levels of TNF−α and IL−4 mRNAs were determined by reverse transcription polymerase chain reaction (RT−PCR) using a PTC−100 programmable thermal controller (MJ Research, Inc., Watertown, MA, USA). All primers were designed using Primer 3 (ver 4.0), an online primer design software (http://primer3.ut.ee/). The primer sequences were as follows: glyceraldehyde−3−phosphate dehydrogenase (GAPDH): forward (F) 5′-AACTTTGGCATTGTGGAAGG-3′, reverse (R) 5′-ACACATTGGGGGTAGGAACA-3′; TNF−α: F 5′-GCAGAAGAGGCACTCCCCCA-3′, R 5′-GATCCATGCCGTTGGCCAGG-3′; IL−4: F 5′-TCAACCCCCAGCTAGTTGTC-3′, R 5′-TGTTCTTCGTTGCTGTGAGG-3′. Operating conditions were 94 °C and 30 s for denaturation, 58 °C and 30 s for annealing, 72 °C and 30 s for polymerization, and 32 cycles. The PCR products were separated on 1.0% agarose gels, stained with GelRed^®^ (Biotium, Fremont, CA, USA), and quantified by measurement of their intensities using an image analysis system (i−Max™; CoreBio System Co., Seoul, South Korea). cDNA expression levels were eventually determined by adjusting each band intensity to that of GAPDH.

### 4.10. Toluidine Blue Staining of HMC-1

Toluidine blue was used to stain degranulated cells of HMC-1. The cells were pelleted by centrifugation at 250 g for 10 min and re-suspended in their supernatant at a concentration of 2 × 10^5^ cells/200 µL. Thin smears were prepared on pre-cleaned defatted slides and subsequently air-dried for 30–60 min. Dried smears were fixed with a freshly prepared 1/1 mixture of 96% ethanol and 96% acetone at 4 °C for 30 min and air-dried. Hydrolysis with 0.1 N HCl at 4 °C for 5 min was followed by washing three times with distilled water for 2 min. The cells on the slides were incubated with toluidine blue solution for 2 min at room temperature and washed three times. Finally, the slides were air-dried and mounted. The slides were viewed at 100× magnification.

### 4.11. Tryptase, PKA, and PKC Activities of HMC-1

Tryptase levels in the culture media of HMC-1 were analyzed by measuring tryptase activity using a mast cell degranulation assay kit (Millipore Co., Billerica, MA, USA) according to the manufacturer’s instruction. The assay is based on spectrophotometric detection of the chromophore *p*-nitroaniline (*p*NA) after cleavage of the labeled substrate Tosyl–Gly–Pro–Lys–*p*NA. Free *p*NA was quantified using a microtiter plate reader (VERSAmax Tunable; Molecular Devices, Sunnyvale, CA, USA) at 405 nm. PKA and PKC activities were determined using the MESACUP Protein Kinase Assay Kit (Medical and Biological Laboratories Co., Ltd., Nagoya, Japan) according to the manufacturer’s instructions. Briefly, PKA/PKCs in the cell extracts phosphorylated the synthetic peptides (RFARKGSLRQKNV) bound to the microplate wells, and the phosphorylated synthetic peptides were selectively bound to a biotinylated monoclonal antibody. Peroxidase-conjugated streptavidin was then added to the wells to bind to the biotinylated monoclonal antibody. Subsequent oxidation of the substrate o-phenylenediamine by the peroxidase was quantitated by measuring the absorbance at 492 nm using an ELISA reader (MultiRead 400).

### 4.12. Western Blot Analysis of MAPK Phosphorylation in HMC-1

A fixed amount of whole cell extracts of HMC-1 was electrophoresed on a 10% sodium dodecyl sulfate-polyacrylamide gel electrophoresis (SDS-PAGE) gel and transferred onto a polyvinylidene difluoride membrane. After blocking with 5% skim milk to prevent nonspecific binding of the sample proteins in the extract, the membrane was incubated overnight with primary antibody specific for phosphorylated or total MAPKs (1:1000, Cell Signaling Technology, Inc., Danvers, MA, USA). After washing with buffer solution, the membranes were subsequently incubated with an appropriate secondary antibody conjugated to horseradish peroxidase. Immunoreactive bands were detected using ECL reagents (Thermo Scientific, Rockford, IL, USA) according to the manufacturer’s instructions. A primary antibody for β-actin (1:5000, Sigma-Aldrich Chemical Co.) was used as a control.

### 4.13. Statistical Analysis

All of the data are presented as means ± SEM. Statistical differences between groups were identified using *t*-test, one-way ANOVA and Tukey’s post hoc test. *p*-values of <0.05 were considered statistically significant.

## 5. Conclusions

In the present study, 2 h-IMO stress for 6 days significantly exacerbated TMA-induced atopic dermatitis by 252% in terms of scratching behavior. The treatment of PTW extract significantly restored IMO stress-induced decreases in skin water content, lymph node weight, and serum histamine and immunoglobulin E (lgE) levels as well as IMO stress-exacerbated scratching behavior and skin inflammation in the mouse model of TMA-induced atopic dermatitis. Furthermore, PTW significantly inhibited SP/CRF-stimulated degranulation of HMC-1 cells, subsequent tryptase secretion, and protein kinase A (PKA) activity. PTW also selectively inhibited p38 mitogen-activated protein kinase (MAPK) phosphorylation in SP/CRF-treated HMC-1 cells. Taken together, PTW extract significantly inhibited HMC-1 cell degranulation and alleviated IMO stress-exacerbated atopic dermatitis symptoms by modulating the PKA/p38 MAPK signaling pathway.

## Figures and Tables

**Table 1:** The observed and calculated mass numbers of Ultra performance liquid chromatography (UPLC) peaks of *Polygala tenuifolia* Radix.

Peak No.	* RT (min)	Theoretical Mass (M-H)	Observed Mass (M-H)	Mass Difference (^♣^ mmu)	Identification
1	9.024	517.15625	517.14369	−9.65	Sibricose A5
2	9.322	547.16682	547.15764	−10.86	Sibricose A6
3	10.984	547.16682	547.15764	−7.28	Sibricose A1
4	11.867	405.08270	405.08002	−2.16	Lancerin
5	14.940	567.13552	567.12205	−8.32	Polygalaxanthone III
6	18.224	681.20359	681.19396	−11.14	Tenuifoliside A
7	22.310	767.24037	767.22731	−12.54	Tenuifoliside C
8	22.658	1453.44616	1453.43189	−4.77	Tenuifoliose M
9	23.36	1223.36713	1223.34665	−17.61	Tenuifoliose S
10	23.885	1253.37769	1253.36143	−14.27	Tenuifoliose T
11	26.884	1295.38825	1295.36908	−14.47	Tenuifoliose E
12	31.640	1307.38825	1307.37049	−16.23	Tenuifoliose J Tenuifoliose I
13	33.145	1337.39882	1377.38065	−17.67	Tenuifoliose B Tenuifoliose D
14	33.706	1379.40938	1379.37981	−19.88	Tenuifoliose A
15	41.081	1877.78614	1877.75927	−14.22	Onjisaponin Sg
16	41.965	1761.73880	1761.73259	−18.04	Onjisaponin V
17	42.762	1631.71219	1631.69787	−16.56	Onjisaponin O
18	44.296	1571.69107	1571.66867	−14.00	Senegin III
19	44.77	1685.72276	1685.69958	−10.52	Onjisaponin Ng
20	45.237	1673.72276	1673.70909	−12.42	Polygalasaponin XXXII
21	45.485	1817.76501	1817.74989	−18.96	Onjisaponin J

* RT: retention time; ^♣^ mmu: milli mass unit.
